# World Health Organization class-independent risk categorization in mastocytosis

**DOI:** 10.1038/s41408-019-0189-5

**Published:** 2019-03-04

**Authors:** Animesh Pardanani, Terra L. Lasho, Kaaren K. Reichard, Curtis A. Hanson, Ayalew Tefferi

**Affiliations:** 1 Divisions of Hematology, Departments of Internal Medicine and Laboratory Medicine, Mayo Clinic, Rochester, MN USA; 2 Divisions of Hematopathology, Departments of Internal Medicine and Laboratory Medicine, Mayo Clinic, Rochester, MN USA

The 2016 World Health Organization (WHO) system for classification of hematopoietic neoplasms organizes systemic mastocytosis (SM) into five prognostically-relevant morphological/clinical categories: indolent (ISM), smouldering (SSM), SM with an associated hematological neoplasm (SM-AHN), aggressive (ASM), and mast cell leukemia (MCL)^1^. ASM, SM-AHN, and MCL are also referred to as “advanced” SM, in order to underline their significantly worse survival, compared to patients with ISM or SSM^2,3^. The WHO system employed a number of clinical and bone marrow morphological parameters, in order to distinguish among these subcategories: presence or absence of the so-called “B” (i.e., high mast cell burden and serum tryptase level, evidence of non-mast cell morphologic dysplasia or myeloproliferation, organomegaly without functional consequence) or “C” (i.e., cytopenia(s) related to mast cell infiltration, organomegaly with functional consequence, skeletal involvement with large osteolytic lesions, malabsorption with weight loss secondary to gastrointestinal mast cell infiltrates) findings; presence or absence of an associated hematological neoplasm; and presence or absence of criteria for MCL (i.e., ≥20% mast cells on bone marrow aspirate smears)^1^. Accordingly, the presence of a concurrent associated non-mast cell hematological neoplasm defines SM-AHN while the presence of C findings distinguishes ASM from ISM/SSM; the presence of ≥2 B findings distinguishes SSM from ISM.

Despite the above-elaborated commendable effort by the WHO, inter-category distinctions in SM are not always clear-cut and are subject to variable interpretation. The issue is particularly relevant when distinguishing SSM from ISM and ASM, and determining the presence or absence of subtle morphologic features that warrant the diagnosis of SM-AHN. In this regard, recent studies have unveiled easily accessible and objective risk factors in SM as potential surrogates for specific WHO class assignment. In particular, based on 580 consecutive SM patients seen at the Mayo Clinic between 1968 and 2015, we recently developed two contemporary risk models, referred to as the Mayo Alliance prognostic systems (MAPS) for SM^4^: one risk model was based on clinical variables only, including age >60 years, advanced SM vs ISM/SSM, platelet count <150 × 10^9^/l, hemoglobin level below sex-adjusted normal and increased serum alkaline phosphatase (ALP) level; the second model included adverse mutations (e.g., *ASXL1*, *RUNX1*, and *NRAS*) as an independent risk factor. In the current study, we used the same study population used in developing the aforementioned MAPS models^4^, to examine the potential for developing a risk model that does not include WHO class assignment.

The current study was approved by the Mayo Clinic institutional review board. Diagnoses of SM and its subcategories were confirmed by both clinical and bone marrow examinations, as per 2016 WHO criteria^1^. Previously described methods were used for next-generation sequencing (NGS)^5^, which was performed in a subset of the study population. Statistical analyses considered clinical and laboratory data collected at the time of initial diagnosis. Cox regression analysis was applied in order to identify risk factors for survival. The Kaplan–Meier method was used to construct time-to-event curves, which were compared by the log-rank test. *P* values of <0.05 were considered significant. Hazard ratio (HR)-based risk point allocation was employed in order to develop the new WHO class-independent risk model and predictive accuracy was compared to those of MAPS-SM^4^, using Akaike Information Criterion (AIC) and area under the curve (AUC) estimates; the latter were obtained from logistic regression analysis of survival prediction at 5 years. The JMP^®^ Pro 13.0.0 software from SAS Institute, Cary, NC, USA, was used for all calculations.

580 consecutive patients with SM (median age 55 years; range 18–88 years; 52% males) were considered; WHO class assignment included ISM in 291 (50%) patients, ASM in 85 (15%), SM-AHN in 199 (34%), and MCL in 5 (1%). Clinical and laboratory features at presentation are outlined in supplementary table 1 (previously published);^4^ anemia, defined by hemoglobin below the lower limit of the sex-adjusted reference range, was present in 41% of the patients, platelet count <150 × 10^9^/l in 26%, serum albumin <3.5 g/dl in 22%, and increased serum ALP in 54%. Median follow-up was 34 months with 239 (41%) deaths documented. Cytogenetic information was available in 342 cases, including 51 (15%) with abnormal karyotype. NGS-derived mutation information was available in 150 cases (Supplementary table 1).

Supplementary table 2 (also previously published)^4^ lists clinical and laboratory parameters that were significant for survival, in univariate analysis. Subsequent multivariable analysis that did not include WHO class assignment or genetic information identified the following as independent risk factors in 380 informative patients: age >60 years (HR 2.7, 95% CI 2.0–3.7), platelet count <150 × 10^9^/l (2.8, 2.0–3.8), hemoglobin below the lower limit of the sex-adjusted reference range (2.6, 1.9–3.7), increased serum ALP (2.1, 1.5–3.1), and serum albumin <3.5 g/dl (1.5, 1.1–2.0). HR-based risk point allocation assigned 2 points for age, platelet count, and hemoglobin level and one point each for serum ALP and albumin levels, which resulted in a five-tiered new risk model, which we will henceforth refer to as WHO class-independent MAPS (Fig. 1a). The particular model was comparable in its predictive accuracy to that of the recently reported MAPS model^4^, which included WHO class assignment (Fig. 1b); the respective AIC values were 1805 vs 1796 and AUC levels 0.87 vs 0.88.

**Fig. 1:**
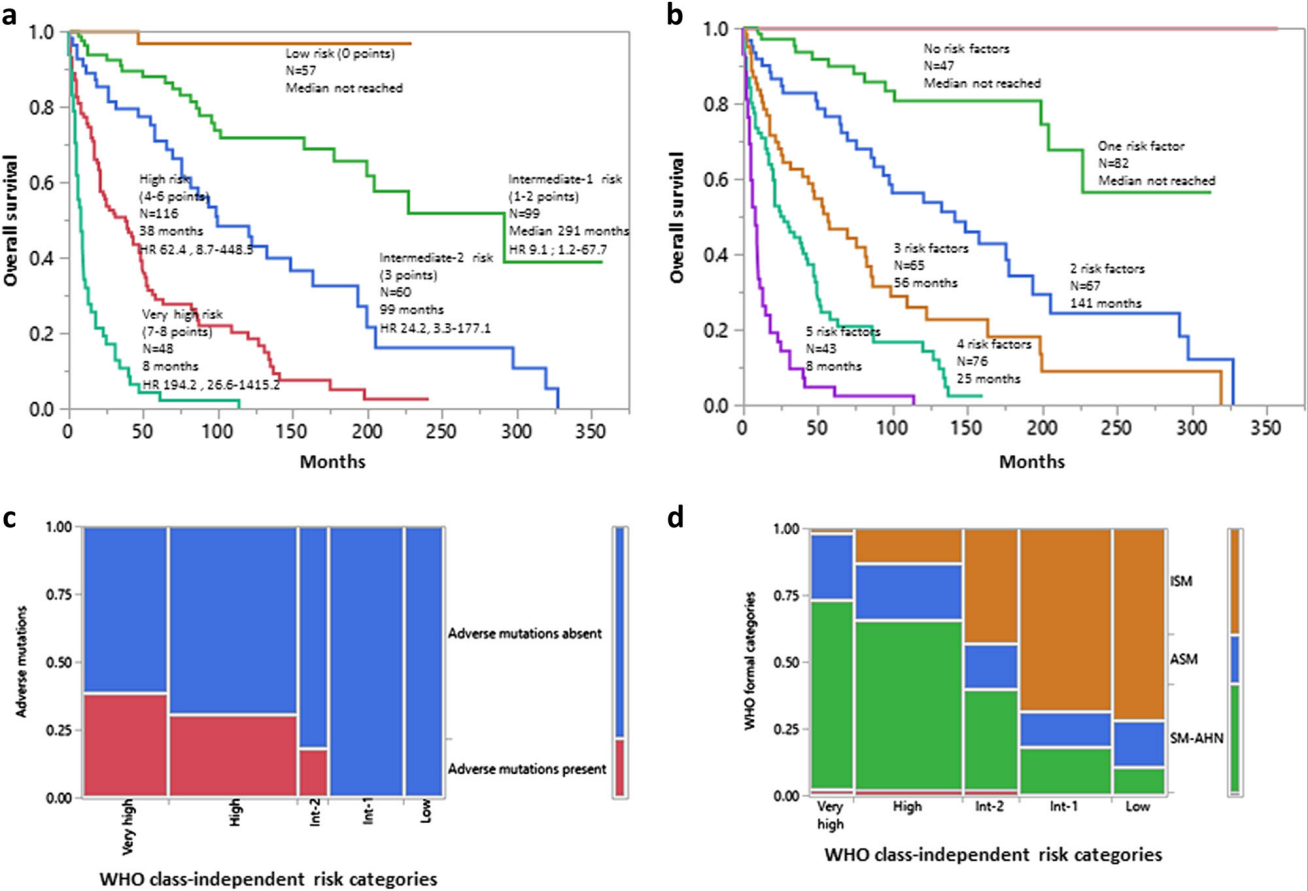
**a** A new risk model for systemic mastocytosis (SM) that does not include World Health Organization (WHO) class assignment or genetic information (*n* = 380); *risk factors: i*) age >60 years (2 points), ii) platelets <150 × 10^9^/l (2 points), iii) anemia below sex-adjusted normal (2 points), iv) serum alkaline phosphatase above normal range (1 point), and v) serum albumin <3.5 g/dl (1 point). **b** The same group of 380 patients stratified by the Mayo Alliance Prognostic System, which includes WHO class assignment; risk factors: i) age >60 years, ii) platelets <150 × 10^9^/l, iii) anemia below sex-adjusted normal, iv) serum alkaline phosphatase above normal range, and v) advanced vs indolent/smouldering SM. **c** Distribution of adverse mutations among the new WHO class-independent risk categories. **d** Distribution of formal WHO categories of SM among the new WHO class-independent risk categories: *ISM* indolent SM, includes smouldering SM, *ASM* aggressive SM, *SM-AHN* SM associated with another non-mast cell hematologic neoplasm

In the context of the new risk model, there was limited additional prognostic information from adverse mutations, which appeared to almost exclusively cluster with very high and high risk disease (Fig. 1c); among 129 informative patients, adverse mutations were seen in a total of 28 patients, 26 of whom were in the very high (*n* = 12) or high (*n* = 14) risk category while the remaining two cases belonged to intermediate-2 risk category. Also, there was poor concordance between the new risk model and formal WHO subcategories of SM (Fig. 1d). On a broader level, risk assignment between the two models displayed better concordance for ISM/SSM and SM-AHN patients, who were in large part distributed as expected in lower (low and intermediate-1/2) and higher (high/very high and intermediate-2) risk groups, respectively, according to the new model (Fig. 1d). In contrast, risk assignment of ASM patients appeared significantly discrepant, with 14–36% of patients being assigned to each risk category in the new model (Fig. 1d).

We conclude that (i) the WHO class-independent MAPS model provides proof-of-concept regarding feasibility of risk categorization of SM patients independent of their WHO class assignment; (ii) poor concordance between the two models indicates that the WHO class-independent MAPS model provides a novel approach to risk assessment of SM patients; (iii) while the classification of some SM-AHN patients in lower risk WHO class-independent MAPS categories can be postulated to reflect presence of indolent AHN’s (e.g., low-grade lymphomas or chronic myeloproliferative neoplasms), the broad distribution of ASM patients across all risk groups in the new model is more challenging to explain. We speculate that not all ‘C’ findings have a similarly adverse prognostic impact on survival, or, alternatively, the prognostic impact of some ‘C’ findings but not others may be mitigated by SM-directed therapies such as 2-chlorodeoxyadenosine or interferon-α; and (iv) the performance of the WHO class-independent MAPS model will require validation, including its assessment in the setting of newer targeted therapies for SM such as midostaurin and avapritinib.

## Supplementary information

Supplementary Table 1

Supplementary Table 2
