# Supplementary material for: World Health Organization class-independent risk categorization in mastocytosis
Source: Blood Cancer J. 2019 Mar 4;9(3):29. doi: 10.1038/s41408-019-0189-5 (PMC6399221; doi:10.1038/s41408-019-0189-5)
Supplement: Supplementary file 1 — Supplementary Table 1 [file 41408_2019_189_MOESM1_ESM.docx]

**Table 1**: Presenting clinical and laboratory characteristics of 580 patients with systemic mastocytosis (SM) seen at the Mayo Clinic between 1968 and 2015

| **Variables** | **All patients**  **N=580** | **Indolent/**  **smouldering SM**  **N=291** | **Advanced SM**  **N=289** | **P value** | **Aggressive SM**  **N=85** | **SM-AHN**  **N=199** | **Mast cell leukemia**  **N=5** | **P value** |
| --- | --- | --- | --- | --- | --- | --- | --- | --- |
| Median age in years (range) | 55 (18-88) | 48 (19-87) | 64 (18-88) | **<.001** | 61 (32-86) | 65 (18-88) | 57 (45-74) | 0.08 |
| Age >60 years; *n* (%) | 235 (41) | 65 (22) | 170 (59) | **<.001** | 44 (52) | 124 (62) | 2 (40) | 0.2 |
| Males; *n* (%) | 301 (52) | 127 (44) | 174 (60) | **<.001** | 38 (45) | 132 (66) | 4 (80) | **0.002** |
| Hemoglobin, g/dl, median (range) | 13.1 (5.1-17.4) | 13.9 (8.1-17.2) | 11.1 (5.1-17.4) | **<.001** | 12.3 (5.1-17) | 10.8 (5.2-17.4) | 10.9 (9.5-11.5) | **0.03** |
| Anemia sex adjusted; *n* (%)  *“N” Evaluable=574* | 236 (41) | 41 (14)  N Evaluable=285 | 195 (67) | **<.001** | 44 (52) | 146 (73) | 5 (100) | **<.001** |
| Leukocyte count x 10^9^/l, median (range)  *“N” Evaluable=573* | 7.1 (0.7-87.2) | 6.5 (1.6-22.2)  N Evaluable=284 | 8.3 (0.7-87.2) | **<.001** | 7.3 (1.7-37.1) | 9 (0.7-87.2) | 4.5 (3.8-7) | **0.004** |
| Platelet count x 10^9^/l, median (range)  *“N” Evaluable=567* | 229 (2-1625) | 257.5 (39-563)  N Evaluable=280 | 157 (2-1625)  N Evaluable=287 | **<.001** | 218.5 (19-570)  N Evaluable=84 | 134.5 (2-1625)  N Evaluable=198 | 145 (54-241) | **<.001** |
| Platelet count <150 x 10^9^/l; *n* (%)  *“N” Evaluable=567* | 149 (26) | 13 (5)  N Evaluable=280 | 136 (47)  N Evaluable=287 | **<.001** | 23 (27)  N Evaluable=84 | 110 (56)  N Evaluable=198 | 3 (60) | **<.001** |
| Urticaria pigmentosa; *n* (%)  *“N” Evaluable=577* | 234 (41) | 162 (56)  N Evaluable=288 | 72 (25) | **<.001** | 32 (38) | 40 (20) | 0 (0) | **0.003** |
| Mast cell mediator symptoms; *n* (%)  *“N” Evaluable =349* | 162 (46) | 106 (69)  N Evaluable=153 | 56 (29)  N Evaluable=196 | **<.001** | 14 (27)  N Evaluable=52 | 40 (29)  N Evaluable=140 | 2 (50)  N Evaluable=4 | 0.6 |
| Serum tryptase ng/ml; median (range)  N Evaluable=105  Serum tryptase <20; *n* (%)  Serum tryptase 20-200; *n* (%)  Serum tryptase >200; *n* (%)  *“N” Evaluable=402* | 73.7 (7.68-1450)  87 (22)  266 (66)  49 (12) | 21.75(11.4-1410)  N Evaluable=37  62 (27)  156 (68)  11 (5)  N Evaluable=229 | 115.5 (7.68-1450)  N Evaluable=68  25 (14)  110 (64)  38 (22)  N Evaluable=173 | **0.01**  **<.001** | 180 (15-1450)  N Evaluable=19  5 (8)  40 (68)  14 (24)  N Evaluable=59 | 99.35 (7.68-1360)  N Evaluable=48  19 (17)  69 (62)  23 (21)  N Evaluable=111 | 1110 (1110-1110)  N Evaluable=1  1 (33)  1 (33)  1 (33)  N Evaluable=3 | 0.2  0.4 |
| BM mast cell %; median (range)  *“N” Evaluable=131*  BM mast cell % <5%; *n* (%)  BM mast cell % 5% to 10%; *n* (%)  BM mast cell % 11% to 50%; *n* (%)  BM mast cell % >50%; *n* (%)  *“N” Evaluable= 432* | 12.5 (5-70)  31 (7)  211 (49)  144 (33)  46 (11) | 10 (5-50)  N Evaluable=43  26 (12)  124 (56)  58 (26)  12 (5)  N Evaluable=220 | 15 (5-70)  N Evaluable=88  5 (2)  87 (41)  86 (40)  34 (16)  N Evaluable=212 | 0.1  **<.001** | 18.75 (5-70)  N Evaluable=22  2 (3)  26 (38)  23 (34)  17 (25)  N Evaluable=68 | 15 (5-70)  N Evaluable=65  3 (2)  61 (44)  63 (45)  13 (9)  N Evaluable=140 | 70 (70-70)  N Evaluable=1  0 (0)  0 (0)  0 (0)  4 (100)  N Evaluable=4 | 0.4  **<.001** |
| Palpable hepatomegaly; *n* (%)  *“N” Evaluable=579* | 119 (21) | 25 (9) | 94 (33)  N Evaluable=288 | **<.001** | 28 (33) | 65 (33)  N Evaluable=198 | 1 (20) | 0.8 |
| Palpable splenomegaly; *n* (%)  *“N” Evaluable=578* | 179 (31) | 33 (11)  N Evaluable=290 | 146 (51)  N Evaluable=288 | **<.001** | 36 (42) | 107 (54)  N Evaluable=198 | 3 (60) | 0.2 |
| Serum albumin, g/dl; median (range)  “*N” Evaluable=389* | 3.9 (2-5.1) | 4 (2.9-5.1)  N Evaluable=157 | 3.8 (2-4.9)  N Evaluable=232 | **<.001** | 3.8 (2-4.9)  N Evaluable=71 | 3.8 (2-4.8)  N Evaluable=157 | 3.85 (3.1-4.4)  N Evaluable=4 | 0.6 |
| Serum albumin <3.5 g/dl; *n* (%)  *“N” Evaluable=389* | 87 (22) | 18 (11)  N Evaluable=157 | 69 (30)  N Evaluable=232 | **<.0001** | 18 (25)  N Evaluable=71 | 50 (32)  N Evaluable=157 | 1 (25)  N Evaluable=4 | 0.6 |
| Serum ALP, U/l; median (range)  *“N” Evaluable=547* | 124 (19-3680) | 92 (30-1957)  N Evaluable=269 | 178.5 (19-3680)  N Evaluable=278 | **<.001** | 195 (33-2004)  N Evaluable=82 | 170 (19-3680)  N Evaluable=191 | 240 (139-1423) | 0.4 |
| Serum ALP >UNL; *n* (%)  *“N” Evaluable=547* | 296 (54) | 101 (38)  N Evaluable=269 | 195 (70)  N Evaluable=278 | **<.001** | 58 (71)  N Evaluable=82 | 132 (68)  N Evaluable=191 | 5 (100) | 0.3 |
| *KITD816V; n* (%)  *“N” Evaluable=357* | 279 (78) | 141 (82)  N Evaluable=172 | 138 (75)  N Evaluable=185 | 0.09 | 41 (87)  N Evaluable47 | 97 (71)  N Evaluable=137 | 0 (0)  N Evaluable=1 | **0.01** |
| *ASXL1* mutated; *n* (%)  *“N” Evaluable=150* | 25 (17) | 0 (0)  N Evaluable=43 | 25 (23)  N Evaluable=107 | **<.001** | 4 (15)  N Evaluable=26 | 21 (26)  N Evaluable=80 | 0 (0)  N Evaluable=1 | 0.4 |
| *RUNX1* mutated; *n* (%)  *“N” Evaluable=150* | 5 (3) | 0 (0)  N Evaluable43 | 5 (5)  N Evaluable=107 | 0.15 | 0 (0)  N Evaluable=26 | 5 (6)  N Evaluable=80 | 0 (0)  N Evaluable=1 | 0.4 |
| *NRAS* mutated; *n* (%)  *“N” Evaluable=150* | 4 (3) | 0 (0)  N Evaluable=43 | 4 (4)  N Evaluable=107 | 0.19 | 0 (0)  N Evaluable=26 | 3 (4)  N Evaluable=80 | 1 (100)  N Evaluable=1 | **<.001** |
| Adverse mutations; *n* (%)  *“N” Evaluable=150* | 31 (21) | 0 (0)  N Evaluable=43 | 31 (29)  N Evaluable=107 | **<.001** | 4 (15)  N Evaluable=26 | 26 (33)  N Evaluable=80 | 1 (100)  N Evaluable=1 | 0.07 |
| Abnormal karyotype; *n* (%)  *“N” Evaluable=348* | 53 (15) | 8 (7)  N Evaluable=142 | 45 (22)  N Evaluable=206 | **<.001** | 4 (8) | 40 (26) | 1 (50)  N Evaluable=2 | **0.02** |
| Median follow-up in months (range) | 34 (0-496) | 51 (0-357) | 23 (0-496) | **<.001** | 35 (0-496) | 20 (0-291) | 2 (1-30) | 0.06 |
| Deaths; *n* (%) | 239 (41) | 44 (15) | 195 (67) | **<.001** | 42 (49) | 150 (75) | 3 (60) | **<.001** |
| Leukemic transformations; *n* (%) | 9 (2) | 0 (0) | 9 (3) | **<.001** | 1 (1) | 8 (4) | 0 (0) | 0.4 |

***Abbreviations:***

SM; systemic mastocytosis

SM-AHN; Systemic mastocytosis with an associated hematological neoplasm

ALP; alkaline phosphatase

UNL; Upper Normal Limit

BM; bone marrow
