# Supplementary material for: World Health Organization class-independent risk categorization in mastocytosis
Source: Blood Cancer J. 2019 Mar 4;9(3):29. doi: 10.1038/s41408-019-0189-5 (PMC6399221; doi:10.1038/s41408-019-0189-5)
Supplement: Supplementary file 2 — Supplementary Table 2 [file 41408_2019_189_MOESM2_ESM.docx]

**Table 2**: Univariate analysis of risk factors for overall survival among 580 patients with systemic mastocytosis

| **Variables** | **All patients**  **Univariate**  **P value**  **N=580** | **Indolent SM**  **Univariate**  **P value**  **N=291** | **Advanced SM**  **Univariate**  **P value**  **N=289** |
| --- | --- | --- | --- |
| Median age in years (range) | **<.001** | **<.001** | **<.001** |
| Age >60 years; *n* (%) | **<.001** | **<.001** | **<.001** |
| Males; *n* (%) | **<.001** | 0.4 | 0.17 |
| Anemia sex adjusted; *n* (%)  *“N” Evaluable=574* | **<.001** | **<.001**  N Evaluable=285 | **<.001** |
| Leukocyte count x 10^9^/l, median (range)  *“N” Evaluable=573* | **<.001** | 0.3  N Evaluable=284 | **0.03** |
| Platelet count x 10^9^/l, median (range)  *“N” Evaluable=567* | **<.001** | 0.4  N Evaluable=280 | **<.001**  N Evaluable=287 |
| Platelet count <150 x 10^9^/l; *n* (%)  *“N” Evaluable=567* | **<.001** | 0.08  N Evaluable=280 | **<.001**  N Evaluable=287 |
| Urticaria pigmentosa; *n* (%)  *“N” Evaluable =577* | **<.001** | 0.7  N Evaluable=288 | **<.001** |
| Mast cell mediator symptoms; *n* (%)  *“N” Evaluable =349* | **<.001** | **0.009**  N Evaluable=153 | 0.68  N Evaluable=196 |
| Serum tryptase ng/ml; median (range)  *“N” Evaluable=105* | **<.001** | **0.003**  N Evaluable=37 | **0.04**  N Evaluable=68 |
| BM mast cell %; median (range)  *“N” Evaluable=131* | **0.002** | **0.01**  N Evaluable=43 | 0.11  N Evaluable=88 |
| Palpable hepatomegaly; *n* (%)  *“N” Evaluable=579* | **<.001** | **0.03** | **0.01**  N Evaluable=288 |
| Palpable splenomegaly; *n* (%)  *“N” Evaluable=578* | **<.001** | **0.006**  N Evaluable=290 | **<.001**  N Evaluable=288 |
| Serum albumin, g/dl; median (range)  “*N” Evaluable=389* | **<.001** | 0.4  N Evaluable=157 | **<.001**  N Evaluable=232 |
| Serum albumin <3.5 g/dl; *n* (%)  *“N” Evaluable=389* | **<.001** | 0.2  N Evaluable=157 | **0.01**  N Evaluable=232 |
| Serum ALP, U/l; median (range)  *“N” Evaluable=547* | **<.001** | **<.001**  N Evaluable=269 | **<.001**  N Evaluable=278 |
| Serum ALP >UNL; *n* (%)  *“N” Evaluable=547* | **<.001** | **0.001**  N Evaluable=269 | **<.001**  N Evaluable=278 |
| *KITD816V; n* (%)  *“N” Evaluable=357* | 0.4 | 0.1  N Evaluable=172 | 0.16  N Evaluable=185 |
| *ASXL1* mutated; *n* (%)  *“N” Evaluable=150* | **<.001** | No adverse  mutations | **<.001**  N evaluable=107 |
| *RUNX1* mutated; *n* (%)  *“N” Evaluable=150* | **0.03** | No adverse  mutations | 0.05  N evaluable=107 |
| *NRAS* mutated; *n* (%)  *“N” Evaluable=150* | **0.002** | No adverse  mutations | **<.001**  N evaluable=107 |
| Adverse mutations; *n* (%)  *“N” Evaluable=150* | **<.001** | No adverse  mutations | **<.001**  N evaluable=107 |
| Abnormal karyotype; *n* (%)  *“N” Evaluable=53* | **<.001** | 0.3  N Evaluable=8 | **<.001**  N Evaluable=45 |

***Abbreviations:***

SM; systemic mastocytosis

SM-AHN; Systemic mastocytosis with an associated hematological neoplasm

ALP; alkaline phosphatase

UNL; Upper Normal Limit

BM; bone marrow
